# Endométrite tuberculeuse: à propos d’un cas et revue de la littérature

**DOI:** 10.11604/pamj.2013.16.94.3430

**Published:** 2013-11-12

**Authors:** Kamilia Laabadi, Fatima Zohra Fdili Alaoui, Sofia Jayi, Hakima Bouguern-Hekmat Chaara, Moulay Abdilah Melhouf

**Affiliations:** 1 Service de gynécologie obstétrique II, CHU Hassan II, Université Mohamed Ben Abdellah, Fès, Maroc

**Keywords:** Tuberculose génitale, endomètrite, infertilité, genital tuberculosis, endometritis, infertility

## Abstract

La tuberculose constitue encore un problème de santé préoccupant, aussi bien dans les pays en voie de développement que dans les pays développés, en partie du fait de l’éclosion de l’épidémie mondiale de l’infection par le virus de l’immunodéficience humaine. Dans sa localisation génitale assez rare (6-10% des localisations tuberculeuses), la tuberculose pose des problèmes diagnostiques. Les symptômes communément rencontrés sont non spécifiques, ce qui contribue au retard thérapeutique et majore le risque d’infertilité qui reste la séquelle quasi inéluctable. A travers une observation d’endométrite tuberculeuse et une revue de la littérature nous étudions les aspects épidémio-cliniques, thérapeutiques et pronostiques de cette localisation tuberculeuse.

## Introduction

La tuberculose constitue un problème de santé publique aussi bien dans les pays en voie de développement que dans les pays développés, en partie du fait de l’éclosion de l’épidémie mondiale de l’infection par le virus de l’immunodéficience humaine.

La tuberculose et plus précisément la tuberculose génitale sévit encore à l’état endémique au Maroc, malgré les moyens mis en œuvre pour son éradication, en particulier la vaccination antituberculeuse systématique à la naissance et le traitement anti-bacillaire codifié et délivré gratuitement dans des structures spécialisées.

Dans sa localisation génitale, la tuberculose pose des problèmes diagnostiques. Les symptômes communément rencontrés sont non spécifiques, ce qui contribue au retard thérapeutique et majore le risque d’infertilité qui reste la séquelle quasi inéluctable.

## Patient et observation

Il s´agit de Mme KM âgée de 27 ans, traitée pour tuberculose pulmonaire à l´âge de 13 ans (mise sous traitement antibacillaire pendant 8 mois), mariée depuis 4 ans, avec notion d’infertilité, qui a consulté dans notre structure pour prise en charge d’une aménorrhée primaire, chez qui l´examen clinique était sans particularité notamment des caractères sexuels secondaires bien développés.

**Figure 1:**
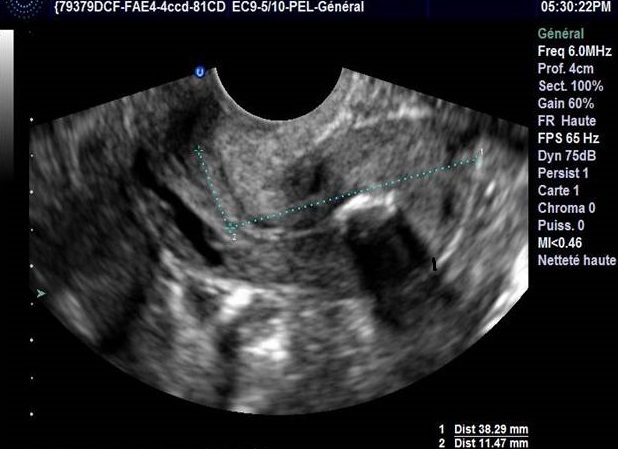
Aspect échographique montrant la ligne d’interface suivie jusqu’au fond où on note la présence d’une image hyperéchogène avec cône d’ombre postérieur (4,58mm)

La patiente a bénéficié d´une échographie pelvienne objectivant un utérus de taille normale, la ligne d’interface était suivie jusqu’au fond ou on note la présence d’une image hyperéchogène avec cône d’ombre post (4,58 mm), les ovaires étaient sans particularités (Figure 1). L’hystérosonographie a objectivé une synéchie utérine totale avec un obstacle infranchissable au niveau de la région cervicale utérine.

**Figure 2:**
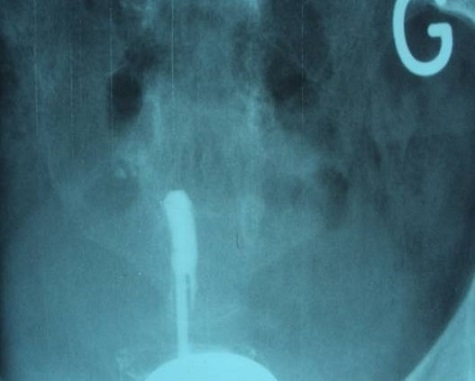
Aspect hystérosalpingographique en faveur d’une synéchie utérine totale, seul 1,5 cm de cette portion cervicale utérine

**Figure 3:**
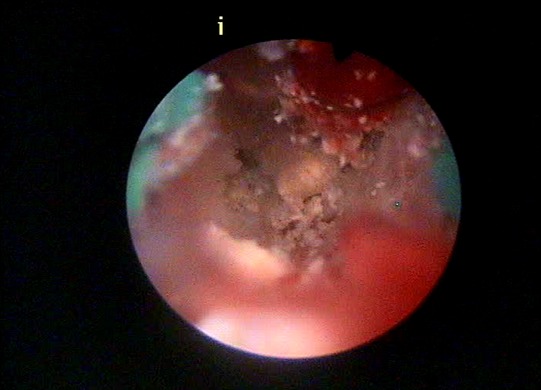
Aspect hystéroscopique de la synéchie utérine infranchissable

**Figure 4:**
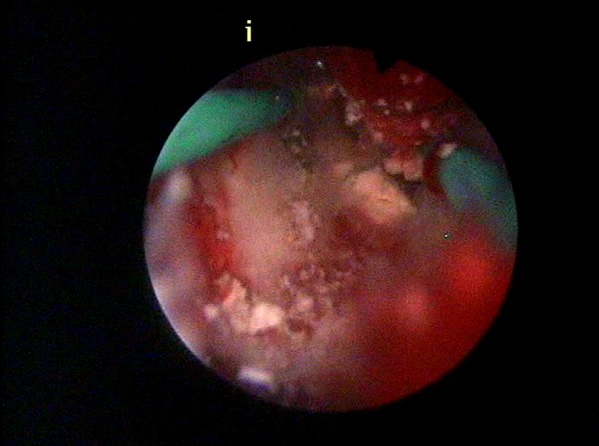
À la résection hystéroscopique, issue d’un enduit blanchâtre faisant évoquer en premier du caséum

L’hystérosalpingographie était en faveur d’une synéchie utérine totale, seul 1,5 cm de cette portion cervicale a été opacifié (Figure 2). L´hystéroscopie diagnostique a objectivé une synéchie utérine infranchissable. Une biopsie a été réalisée avec issue d´un enduit blanchâtre faisant évoquer en premier du caséum. (Figure 3, Figure 4). L´étude histologique était compatible avec la présence d’un granulome associé à un caséum. L´urographie intraveineuse n´a pas objectivé d´autres lésions urinaires. Le bilan phtysiologique, fait d’une radiographie du thorax, recherche de bacille de Kock dans les crachats et intradermoréaction, est revenu négatif.

La patiente a reçu le traitement anti bacillaire à base 2RHZE/6RH; les deux premiers mois: Rifampicine, Isoniazide, Pyrazinamide et Ethambutol relayé pendant 4 mois par Isoniazide et Rifampicine.

## Discussion

La tuberculose est toujours un sujet d’actualité avec environ 9 millions de personnes atteintes chaque année et un million et demi enregistré annuellement [1]. En France, les taux restent stables depuis 1998 avec environ 6 300 cas par an. Les formes pulmonaires isolées ou associées représentent 72% des cas [2]. Au Maroc, durant l’année 2007, 500 à 1.000 décès dus à la tuberculose et 25.562 nouveaux cas ont été répertoriés, soit une stagnation de l’incidence de la maladie [1, 3]. Dans les pays en voie de développement, la tuberculose reste fortement endémique.

Alors que la tuberculose pulmonaire, forme la plus fréquente d’atteinte bacillaire, représente un souci permanent de santé publique, la forme génitale de cette affection demeure sous-estimée et peu citée, cette situation expliquant le caractère généralement tardif du diagnostic [4, 5]. Cette forme représente 6 à 10% de l’ensemble des localisations tuberculeuses [6]. Malgré la rareté de cette forme clinique, on retrouve quelques cas décrits majoritairement dans les pays non-industrialisés

La tuberculose génitale féminine est caractérisée par la fréquence des formes latentes, inapparentes. Elle est toujours secondaire et succède soit: à une dissémination par voie hématogène à partir d’un foyer tuberculeux initial, avec une atteinte initiale des trompes (100% des cas) réalisant un tableau de salpingite à partir de laquelle l’infection progresse vers les autres organes génitaux (endomètre dans 50% des cas, ovaires dans 20% des cas, col utérin dans 5%, vagin et vulve dans 8% des cas); soit à une contamination par voie lymphatique à partir des ganglions pelviens ; de rares cas secondaires à une inoculation directe par contage vénérien ont été rapportés par Weinstein [1, 2, 6].

La tuberculose pelvienne touche de façon prédominante la femme jeune, en pleine activité génitale mais il existe des formes déclarées en péri- ou postménopause [1, 2], ces formes sont dues le plus souvent à une longue période de latence de la maladie, plus rarement à une atteinte tuberculeuse tardive. Notre patiente est jeune de 27 ans avec antécédents de tuberculose pulmonaire dans l’enfance.

Les circonstances de découverte de la tuberculose génitale féminine sont très variées. Si, autrefois, les formes classiques représentées par les formes ascitiques et les pelvipéritonites étaient les plus fréquentes, les formes actuelles sont plus pauci-symptomatiques et souvent découvertes à la suite d’une infertilité primaire ou secondaire (44%) [1].

En effet, la stérilité primaire (comme le cas de notre patiente) est le motif de consultation le plus fréquent dans les principales publications (60%) [6–8]. C’est pourquoi la recherche de l’étiologie tuberculeuse doit être systématique chez les femmes stériles [6]. Les métrorragies et les douleurs pelviennes sont aussi des symptômes fréquents retrouvés dans environ 40% des cas L’aménorrhée primaire ou plus souvent secondaire est un autre motif de consultation, retrouvée dans 5 à 20% des cas [6, 9]. Elle peut être due à une synéchie utérine comme le cas de notre patiente, à des troubles de réceptivité de l’endomètre, ou à l’anovulation due aux adhérences péritonéales [6]. Rarement une tuberculose génitale se manifeste par une masse abdominale, une ascite, ou un abcès tubo-ovarien [1, 10].

L’endométrite tuberculeuse chez la jeune femme est presque toujours associée à la salpingite tuberculeuse contrairement aux femmes ménopausées où souvent l’endométrite est isolée sans atteinte tubaire [1, 11]. L’examen clinique est le plus souvent normal et n’apporte que rarement une aide diagnostique.

Le bilan biologique est d’un intérêt médiocre : vitesse de sédimentation accélérée, lymphocytose, modification des gammaglobulines.

Les techniques d’imagerie ne sont pas spécifiques [1, 12]. La radiographie pulmonaire peut mettre en évidence des séquelles parenchymateuses ou pleurales, moins souvent des lésions évolutives. L’urographie intraveineuse est utile en raison de la fréquence de la tuberculose urinaire associée.

La plupart des auteurs accordent une place de choix à l’hystérosalpingographie. Elle peut montrer des adénopathies pelviennes calcifiées, et des synéchies de la cavité utérine, réalisant un aspect typiquement en doigt de gant ou, si l’utérus est entièrement symphysé, l’opacification de l’endocol ou de l’isthme seulement comme c’est le cas de notre patiente. On peut voir également des sténoses des trompes leur donnant un aspect rigide, des images d’abcès ou d’hydrosalpinx non-caractéristiques. Cependant, deux aspects sont caractéristiques de l’origine tuberculeuse : les synéchies utérines et l’image de passage vasculaire qui donne le classique angiogramme de KIKA retrouvées respectivement dans 30% et 15% des cas [6, 13]. L’image de passage vasculaire est un signe de grande valeur en faveur d’une endométrite tuberculeuse évolutive qu’il faut rapidement traiter. Les lésions tubaires sont plus fréquentes avec prédominance des sténoses. Bouraoui [14] a rapporté 100% des sténoses tubaires dans une série de 49 malades en 1986. L’hystérosalpingographie de notre patiente a objectivé une synéchie utérine avec seulement 1,5 cm du canal cervical opacifié.

En cas d’autres lésions génitales associées, la cœlioscopie peut présenter un intérêt majeur pour le diagnostic, le bilan d’extension et l’appréciation de l’évolutivité.

Le diagnostic de certitude est obtenu par la mise en évidence du *Mycobacterium tuberculosis* soit à l’examen direct microscopique, soit après mise en culture de prélèvements pathologiques. Le matériel analysé est obtenu par curetage biopsique endométrial, ou par laparoscopie voire laparotomie avec parfois hystérectomie [1, 2]. Son isolement, à partir des sécrétions génitales, est toujours exceptionnel malgré l’avènement de la PCR. Oosthuizen et al. [7] ont rapporté une recherche de bacille de Koch positive à partir du sang des règles dans 3,6% parmi une population de 109 femmes stériles. Sfar [15] a rapporté une recherche de bacille de Koch positive à partir des sécrétions cervicales dans 6,2% dans une série de 118 malades.

L’examen histologique des biopsies génitales reste l’examen clé pour confirmer le diagnostic, tout en sachant que la nécrose caséeuse peut manquer dans d’authentiques tuberculoses. Selon Taleb [13] la négativité de l’étude histologique ne permet pas d’écarter la tuberculose, et doit au contraire inciter à la répéter. Dans notre cas on n’a pas pu mettre en évidence la présence de granulome vu l’absence de tissu endométrial au niveau des prélèvements faits au cours de l’hystéroscopie.

Le traitement de la tuberculose génitale féminine est avant tout médical, associant un traitement antituberculeux quadruple (Isoniazide, Rifampicine, Pyrazinamide et Ethambutol) pendant 2 mois relayé par un traitement double (Isoniazide et Rifampicine) pour une durée de 6 mois. La surveillance clinique et paraclinique s’effectue régulièrement tout au long du traitement [1, 2]. Notre patiente a été traitée pour une endométrite tuberculeuse devant le jeune âge, l’ATCDs de tuberculose pulmonaire, l’aménorrhée primaire et l’aspect hystéroscopique (l’aspect de l’enduit blanchâtre évoquant un caséum).

Le traitement chirurgical ne se justifie qu’en présence de lésions volumineuses réagissant peu ou pas au traitement médical [6, 13] : persistance d’une masse annexielle, abcès froid, rechute de la tuberculose endométriale après une année de traitement, persistance de douleurs pelviennes après 3 mois de traitement ou lorsqu’elles n’ont pas totalement disparu au terme d’un an de traitement, métrorragies persistant après guérison anatomique et clinique, fistules qui ne se tarissent pas [1, 13]. La chirurgie devrait être réalisée au moins 6 semaines après le début du traitement antibacillaire car ce dernier réduit le risque des complications per opératoires et facilite l’abord chirurgical [1, 11]

En cas d’endométrite tuberculeuse, les séquelles gynécologiques sont graves, altérant le plus souvent le potentiel de fertilité des patientes [4]. En effet, la survenue d’une grossesse intra-utérine spontanée est exceptionnelle dans la littérature et le risque de grossesse extra-utérine est important [4]. Face à une telle gravité des séquelles gynécologiques, souvent majorées dans nos pays où nous observons des retards du diagnostic et une sous-évaluation des lésions, il nous paraît essentiel de renforcer la prévention par la vaccination systématique et de rechercher systématiquement une localisation génitale en cas de tuberculose pulmonaire dépistée ou lors d’investigations pour stérilité.

## Conclusion

La tuberculose, fréquente dans note pays, s’exprime rarement par une atteinte endométriale. L’incidence réelle est probablement sous-estimée. Son diagnostic est souvent fait rétrospectivement. Il faut savoir l’évoquer devant une stérilité chez une jeune femme ou devant une symptomatologie pelvienne trainante et réaliser facilement des examens cytobactériologiques. L’importance d’un dépistage précoce et systématique chez les femmes présentant des troubles du cycle menstruel ou une stérilité doit être soulignée, tout particulièrement dans les pays en voie de développement.
